# Changing Patient and Public Beliefs About Antimicrobials and Antimicrobial Resistance (AMR) Using a Brief Digital Intervention

**DOI:** 10.3389/fphar.2021.608971

**Published:** 2021-03-31

**Authors:** Amy Hai Yan Chan, Rob Horne, Helen Lycett, Eva Raebel, Jordi Guitart, Emilie Wildman, Karen Ang

**Affiliations:** ^1^ Centre of Behavioural Medicine, School of Pharmacy, UCL, London, United Kingdom; ^2^ School of Pharmacy, University of Auckland, Auckland, New Zealand; ^3^ Spoonful of Sugar Ltd–a UCL-Business Company, London, United Kingdom

**Keywords:** antimicrobial resist ance, antibiotic, behavioural science, digital intervention, treatment beliefs, beliefs about medicines, pragmatic intervention, online intervention

## Abstract

**Background:** A key driver of antimicrobial resistance (AMR) is patient demand for unnecessary antibiotics, which is driven by patients’ beliefs about antibiotics and AMR. Few interventions have targeted beliefs to reduce inappropriate demand.

**Objective:** To examine whether a brief, online algorithm-based intervention can change beliefs that may lead to inappropriate antibiotic demand (i.e. perceptions of antibiotic necessity and lack of concern about antibiotic harm).

**Design:** Pre- and post-intervention study.

**Participants:** Participants were 18 years or older, and residing in the United Kingdom, who self-selected to participate via Amazon mTurk, an online survey plaform, and via research networks.

**Intervention:** Participants were presented with a hypothetical situation of cold and flu symptoms, then exposed to the intervention. The online intervention comprised: 1) a profiling tool identifying individual beliefs (antibiotic necessity, concerns, and knowledge) driving inappropriate antibiotic demand; 2) messages designed to change beliefs and knowledge (i.e. reduce antibiotic necessity, and increase antibiotic concerns and knowledge), and 3) an algorithm linking specific messages to specific beliefs and knowledge.

**Main measures:** The profiling tool was repeated immediately after the intervention and compared with baseline scores to assess change in beliefs. A paired samples *t*-test was used to determine intervention effect.

**Key Results:** A total of 100 respondents completed the study. A significant change in beliefs relating to inappropriate demand was observed after the intervention, with a reduction in beliefs about antibiotic necessity (t = 7.254; *p* < 0.0001), an increase in antibiotic concerns (t = −7.214; *p* < 0.0001), and increases in antibiotic and AMR knowledge (t = −4.651; *p* < 0.0001).

**Conclusion:** This study is the first to demonstrate that patient beliefs about antibiotics and AMR associated with inappropriate demand can be changed by a brief, tailored online intervention. This has implications for the design of future interventions to reduce unnecessary antimicrobial use.

## Introduction

Antimicrobial resistance (AMR) is one of the greatest threats to public health facing us this century (United Nations, 2016) Reducing antibiotic consumption is required to slow the progression of AMR, and unnecessary use of antibiotics, such as given for common cold or flu symptoms, provides an important opportunity to reduce antibiotic consumption (World Health Organization, 2000) To date, most effective interventions to improve antimicrobial use have focused on clinician prescribing behaviour rather than the behaviour or attitudes of patients and the public, (Ranji et al., 2008; Little et al., 2013; McDonagh et al., 2018) yet patient demand is a key driver of AMR. In a recent study, nearly 50% of general practitioners (GPs) prescribed antibiotics in response to patient expectations or demand, despite being uncertain whether or not antibiotics were clinically necessary. (Cole, 2014; Fletcher-Lartey et al., 2016) It is, therefore, imperative that interventions address this; (Davey et al, 2002; Tonkin-Crine et al., 2011) however, few interventions have focused on changing patient demand.

Demand for antibiotics is driven by patient beliefs about their symptoms and antibiotics. Beliefs about personal need for antibiotics, and lack of concern about antibiotic harm and AMR are particularly salient. (Davey et al., 2002; Norris et al., 2013; Gaarslev et al., 2016) Historically, antimicrobial stewardship (AMS) programs have been delivered in hospital, but as most antimicrobials are used in the community, there has been a recent shift in focus to AMS initiatives for primary care. (Sanchez et al., 2016; Hawes et al., 2020) Public campaigns and interventions that are delivered in primary care centres are part of many national plans for AMR, and include health professional education sessions; electronic decision support; and feedback-based prescribing interventions. (Huttner et al., 2014; Huttner et al., 2014; World Health Organization, 2014; Huttner et al., 2019) The evidence base shows that simply providing information about AMR is however ineffective unless it responds to and changes the underlying beliefs that are driving inappropriate demand. (Kelly and Barker, 2016) Recent reviews of AMS interventions recommend that to target AMR effectively, interventions need to be evidence-based, informed by behaviour change or health psychology theory, and target patient demand through shaping public perception and beliefs. (Huttner et al., 2010; Tonkin-Crine et al., 2011; Huttner et al., 2019)

The first step to reduce inappropriate demand is to identify and address the beliefs that are driving the demand. One approach to this is to apply the Necessity Concerns Framework (NCF) which has proved useful in explaining variation in engagement with other prescribed treatments. (Horne et al., 2013a) Studies across a range of different conditions and medical treatments have shown that treatment engagement often relates to how patients judge their personal need for treatment relative to their perceptions of harm. (Horne et al., 2013a; Foot et al., 2016) Several interventions to enhance treatment engagement have applied this approach to improve medication adherence by addressing misplaced doubts about medication necessity and reducing concerns about harm. (Horne et al., 1997; Clifford et al., 2008; Petrie et al., 2012; Chapman et al., 2019; Riaz et al., 2019)

Patient demand for antibiotics can similarly be explained by the NCF, but in a reverse fashion (NCF-R). Inappropriate demand for antibiotics is often driven by erroneous perceptions of high antibiotic need and low concerns about antibiotic harm (McNulty et al., 2013; Gaarslev et al., 2016; Bakhit et al., 2019), contrasting with non-adherence to prescribed medication, which is often related to perceptions of low personal necessity and high concerns about harm. Addressing these misconceptions is essential to reduce unnecessary demand and motivate patients to adopt other, more appropriate, strategies for managing their acute symptoms such as non-pharmacological self-management strategies.

This paper reports on a proof-of-principle study about whether misplaced beliefs about antibiotics driving inappropriate demand (high necessity, low concerns) can be changed using a brief, tailored algorithm-based digital intervention.

## Methods

### Study Design

This study adopted a pre-and post-intervention study design where the effect of the intervention on baseline measures of patient beliefs was evaluated after exposure to the behaviour change intervention.

### Participants

Adults aged 18 years or over and located in the United Kingdom were eligible for inclusion. There were no exclusion criteria, however, as the intervention consisted of behaviour change messages, a moderate level of English proficiency was required to read and understand the messages.

This study aimed to recruit 100 participants. Study participants were sampled via two methods to maximise generalisability of the study findings. The first sample consisted of participants recruited via Amazon Mechanical Turk (mTurk), an online study participant-recruitment platform hosted via Amazon where participants are reimbursed with small monetary rewards for completion of online tasks such as questionnaires or surveys. mTurk has increasingly been used in health research, due to its relatively low costs, and rapid recruitment rates of diverse samples of participants. Studies have demonstrated that findings generated from mTurk samples appear to be largely comparable to those collected via more traditional recruitment means. (Mortensen and Hughes, 2018) Participants were reimbursed USD$4.20 for their participation. Due to the efficiency of this recruitment method, we aimed to recruit 80% of the target sample size via this method.

A second sample was recruited via research and personal networks of the team, which comprised health professionals, academic researchers, and behaviour change consultants. Participants were mailed the online Qualtrics survey link by the research team, and reimbursed with a £5 Amazon voucher for completion of the study. We aimed to recruit the remaining 20% of the target sample via this channel. According to an online review by the United Kingdom NHS Research Ethics Committee, no further ethical approval was deemed necessary for this study as no identifiable data were collected during the study (COREC, 2018).

### Intervention

The intervention was a logic algorithm that tailored information to address patients’ beliefs in a personalised way (PERSIGNIA, working title, © *Professor Rob Horne*). This comprised two separate but related parts. First, participants were invited to complete a profiling questionnaire to identify participant’s beliefs about antibiotics and AMR. Second, participants were exposed to a set of brief messages designed using behavioural change principles that aimed to increase participants’ perceived concerns about AMR while reducing their perceived necessity for antibiotics. The logic algorithm was used to link specific messages to participants’ individual beliefs so that the messages that participants received were tailored according to their responses to the profiling questionnaire.

### Development of the Profiling Questionnaire

The profiling questionnaire identified each individual’s unique necessity beliefs and concerns about antibiotics and AMR. Statements assessing participants’ beliefs about antibiotics and AMR were adapted from the Beliefs about Medicines questionnaire (BMQ), a widely-used and validated questionnaire that identifies individual’s beliefs about treatment. (Horne et al., 1999) The statements were chosen to reflect the beliefs likely to be associated with antibiotic need and perceptions about AMR. These were identified from literature on patient beliefs that drive demand for antibiotics. (McNulty et al., 2013; Norris et al., 2013; Gaarslev et al., 2016) These statements were then reviewed by the research team, which included the original author of the BMQ.

The final questionnaire comprised 17 items: 8 assessing antibiotic necessity beliefs, 3 relating to concerns about antibiotics, and 6 relating to general perceptions about antibiotics and AMR. Some items were worded negatively to reduce response bias and, thus, reversed scored. Questionnaire responses were scored using a 3-point Likert scale (agree, uncertain, disagree). For the necessity subscale, scoring ranged from 8 to 24, with higher scores indicating high necessity for antibiotics. Participants scoring 8 were considered to have an ‘ideal’ target score, as this was the lowest possible score for necessity. For the concerns subscale, scores ranged from 3 to 9, with higher scores indicating higher concerns. The ‘ideal’ score for concerns was 9–indicating highest concerns for use of antibiotics. For the general perceptions subscale, scores ranged from 6 to 18. Higher scores indicated fewer misconceptions (i.e. more accurate beliefs) about antibiotics and AMR. The ‘ideal’ score was 18, the highest possible score, which indicated that the participant did not hold any misconceptions or inaccurate beliefs about antibiotics and AMR.

The intervention aim was to reduce antibiotic necessity (i.e. *reduction* in necessity subscale scores) and increase concerns (i.e. *increase* in concerns subscale) whilst addressing any misplaced perceptions about antibiotics and AMR (i.e. *increase* in accuracy of general perceptions with an increase in scores on the perceptions subscale).

### The Tailoring Algorithm

A logic algorithm was used within the intervention to tailor the behaviour change messages according to participant responses to the profiling questionnaire, an approach that has demonstrated efficacy in previous studies. (Katzer et al., 2018; Chapman et al., 2019) This algorithm was designed so that if a participant’s responses on any of the 17 items indicated a high need for antibiotics, a lack of concern about antibiotic harm and AMR, and/or misconceptions about antibiotics and AMR, they would be exposed to messages designed to address that belief with the aim of addressing any misplaced beliefs driving antibiotic demand. Participants who had scored the ‘ideal’ score i.e. lowest score for baseline necessity beliefs, highest score on concerns and high accuracy scores on misconceptions about antibiotics and AMR were not exposed to the designated messages for that respective belief/ misconception.

### Development of the Behaviour Change Messages

A total of 17 behaviour change messages were developed, based on the NCF-R and principles of behaviour change. (Horne et al., 2013a) Each message corresponded to an item on the profiling questionnaire. The content of the behaviour change messages was informed by previous literature on patient/public attitudes and beliefs about antimicrobials and AMR, and expertise from the research team. Several key target areas were identified that fit the NCF-R – i.e. drivers of a high level of public perceived necessity for treatment, and minimal public concern about antimicrobials and AMR.

### Intervention Testing

The intervention was tested using a hypothetical-scenario-based methodology. This approach was used to increase the accuracy of participant responses by providing contextual information to participants in the form of a scenario or story vignette, to encourage participants to begin thinking about how they would respond and what they would do in the hypothetical situation described (Hughes, 1998) These stories/scenarios encourage participants to enter into a given mind-set (based on the scenario at hand), and are frequently used in the study of perceptions, beliefs and attitudes. After reading the information sheet and providing consent, participants read the following hypothetical scenario: *“Imagine you have been feeling sick with fevers, aching muscles, and general flu-like symptoms along with a chesty cough. You’ve been feeling like this for the last 3 days, and it isn’t getting better.”*

### Primary Outcomes

Primary endpoints of interest were change in beliefs about antibiotic necessity, concerns about harmful effects of antibiotics and AMR, and misconceptions about antibiotics and AMR.

This was evaluated using the 17-item adapted BMQ (i.e. the profiling questionnaire). Participants completed the adapted BMQ immediately after exposure to the messages according to the algorithm. As such, the profiling questionnaire served two purposes–first, to identify participant beliefs about antibiotics and AMR, to allow tailoring of the intervention to the individual’s unique set of beliefs; and second, to evaluate the effect of the intervention on shifting beliefs.

### Data Analyses

The impact of the AMS intervention messages on necessity beliefs, concerns and general perceptions was assessed by calculating the mean score of the questionnaire items pre- and post-intervention and comparing the magnitude and direction of changes for each subscale (necessity, concerns, perceptions), and in aggregate (i.e. to determine overall effect of the intervention). Scores were analysed using descriptive statistics, and a paired samples *t*-test used to determine the effect of the intervention on patient beliefs.

Direction of impact was coded using positive (+), neutral (0) and negative (−) coding for each of the subscales based on the direction of changes in total scores of each subscale pre- and post-intervention. A positive impact indicates change in the desired direction of the intervention (i.e. decreased necessity, increased concerns, increased accuracy of general perceptions); neutral impact indicates no change between pre- and post-intervention, and negative impact indicates change in the opposite direction to what is desired. This impact analysis was conducted for each subscale separately and in aggregate. The aggregate statistic pooled together the effect of the intervention on each of the respective subscales to determine the impact of the intervention overall (see Table 1).

**TABLE 1:** Different possible combinations of effectiveness for each subscale and corresponding coding of aggregate impact which considers overall effectiveness.

All possible combinations			
	Necessity	Concerns	Perceptions about antibiotics antimicrobial resistance
+++	+	+	+
++	+	+	0
	+	0	+
	0	+	+
+	+	0	0
	0	+	0
	0	0	+
	+	−	+
	+	+	−
	−	+	+
0	+	0	−
	+	−	0
	0	+	−
	0	−	+
	−	+	0
	−	0	+
−	−	0	0
	0	−	0
	0	0	−
	−	+	−
	−	−	+
	+	−	−
− −	−	−	0
	−	0	−
	0	−	−
− − −	−	−	−

To further evaluate the impact of the intervention, the following formula was proposed for each subscale score (Necessity beliefs, Concerns and General perceptions) whereby the score was compared to the ‘ideal’ score pre- and post-intervention to quantify the level of impact of the intervention on shifting beliefs closer to the ‘ideal’ score:

$${\text{Impact|}}_{\text{variable}}\left(\text{\%}\right)\;=\text{ 1}-\frac{\left[{\text{Ideal|}}_{\text{score}}-{\text{Post}-\text{intervention|}}_{\text{score}}\right]}{\left[{\text{Ideal|}}_{\text{score}}-{\text{Pre}-\text{intervention|}}_{\text{score}}\right]}\text{x }100$$

This percentage impact captures how far the post-intervention score was from the ‘ideal’ relative to how far the baseline score was from ‘ideal’. This gives an approximate estimation of the intervention impact with 100% being maximum impact, and 0% being no impact at all. This impact statistic was also calculated for each subgroup of participants depending on recruitment method – for mTurk and non-mTurk participants. The percentage considers subscale scores pre- and post-intervention and compares these against the ideal total score.

A paired samples *t*-test was used to compare the total scores of necessity beliefs, concerns and general perceptions scores pre- and post-intervention.

### Data Quality

As a quality assurance check, a minimum study completion time was used to determine response validity. Participants who completed the study in less than 101 s were excluded and a new participant was recruited as a replacement until the target sample size of 100 participants was reached. The threshold of 101 s was determined by measuring the average time it took a researcher to respond to only the profiling questionnaire without exposure to messages (i.e. to mimic the scenario where the profiling tool scores indicated that the participant did not need to receive any messages). This was defined as the fastest possible completion time for the study, and used as an indicator of data quality. Using this approach, tenparticipants were excluded and replaced during the study.

## Results

A total of 100 respondents completed the online study; 81 were recruited via the Amazon mTurk platform (the mTurk sample), and 19 from research networks (the non-mTurk sample). The mean (SD) completion time was 401 (278) s (range 120–1399 s).

### Effect of the Intervention on Beliefs

There were changes overall across all three domains in the Necessity, Concerns and Knowledge scores, with a reduction in perceived necessity for antibiotics, increase in concerns, and increase in scores for general perceptions (Table 2). The mean shift in scores for most items was in the order of magnitude of 0.2–0.5 per item, representing a shift in a ‘third’ to a ‘half’ of a score category on the 3-point Likert scale. In most cases, these shifts in beliefs were movements away from the ‘uncertain’ category (score = 2) towards the ends of the scale (either disagree (score = 1) for Necessity items; agree (score = 3) for Concerns; and disagree (score = 3) for Perception items) (see Table 2).

**TABLE 2:** Mean (SD) change in Necessity, Concerns and Knowledge scores post-intervention (scores out of a maximum of 3, 1 = agree, 2 = uncertain, 3 = disagree).

	Mean score (baseline)	SD (baseline)	Mean score (post-intervention)	SD (post-intervention)	Mean change in scores	SD (change)
**Necessity subscale (higher scores, higher perceived need for antibiotics)**						
I need antibiotics because I feel really ill	1.76	0.79	1.30	0.61	-0.46	0.73
I need antibiotics now because I am not getting any better	1.93	0.87	1.31	0.69	-0.62	0.85
My body can tell me when I need antibiotics	1.80	0.80	1.33	0.64	-0.47	0.77
I need antibiotics because I’ve been ill for more than 3 days	1.54	0.77	1.21	0.52	-0.33	0.79
Getting an antibiotic is proof that I am ill	1.59	0.81	1.27	0.63	-0.32	0.83
Only antibiotics can make me feel better	1.37	0.63	1.15	0.39	-0.22	0.61
It is best to take an antibiotic to be on the safe side	1.36	0.64	1.17	0.45	-0.19	0.61
I can stop the antibiotics once I feel better^a^	2.46	0.83	2.78	0.52	0.32	0.74
Mean change per item–necessity subscale					-0.37	0.74
Overall change–total necessity subscale					-2.29	4.20
**Concerns subscale (higher scores, higher concerns about antibiotics)**						
I am concerned about antibiotic resistance	2.64	0.66	2.84	0.51	0.2	0.57
Antibiotics are harmless^a^	2.56	0.63	2.85	0.44	0.29	0.62
Taking a short course of antibiotics will not cause side effects^a^	2.41	0.67	2.85	0.44	0.44	0.74
Overall mean change–concerns subscale					0.31	0.65
Overall change–total concerns subscale					0.93	1.68
**Perceptions subscale (higher scores, more accurate perceptions about antibiotics and resistance)**						
There is not much I can do to help reduce antibiotic resistance	2.55	0.63	2.78	0.52	0.23	0.69
Using lower doses of antibiotics can help reduce risk of antibiotic resistance	2.11	0.74	2.64	0.64	0.53	0.74
Taking an antibiotic I don’t need will increase the risk of antibiotic resistance^a^	2.74	0.54	2.69	0.66	-0.05	0.78
Antibiotic resistance is when people become resistant to the bacteria	2.71	0.57	2.83	0.49	0.12	0.54
Antibiotic resistance is when bacteria become resistant to the antibiotic^a^	2.84	0.42	2.92	0.34	0.08	0.46
New antibiotics will be developed in the future	1.44	0.52	1.61	0.65	0.17	0.79
Overall mean change–knowledge subscale					0.18	0.67
Overall change–total knowledge subscale					1.08	2.74

^a^ reverse-scored items.

### Impact on Necessity Beliefs

**FIGURE 1:**
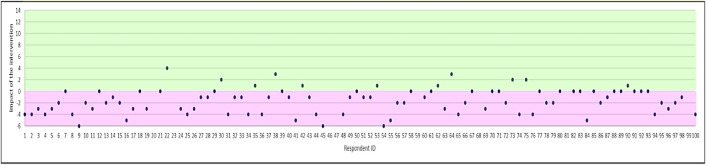
Magnitude of change in Necessity beliefs (difference in total score for the Necessity beliefs subscale). Positive effect of the intervention is shown in purple (i.e. reduction in antibiotic necessity), and negative effect in green.

There was a significant reduction in total Necessity beliefs with scores reducing by 2.29 points after the intervention (*t* = 7.254; *p* < 0.0001). The magnitude of the change in Necessity beliefs for each participant is depicted in Figure 1, which shows the difference between the total score of the necessity items pre- and post-intervention. Negative differences indicate a reduction in necessity beliefs (the desired effect of the intervention). Necessity scores decreased in 67% of respondents, remained the same in 22% of respondents, and increased in 11%.

Overall, the percentage impact of the intervention on Necessity beliefs was 39%; there were not differences between the mTurk and non-mTurk samples (Table 3). Of the items in the Necessity subscale, the intervention had the largest impact shift beliefs relating to “I need antibiotics now because I am not getting any better”. The reverse worded item “I can stop antibiotics once I feel better” was least amenable to change, with an increase in necessity observed for that item.

**TABLE 3:** Percentage impact of the intervention on Necessity beliefs.

Respondent group	Ideal score	Pre-intervention	Post-intervention	% Impact
mTurk (*n* = 81)	648	1138	945	39.4
Non-mTurk (*n* = 19)	152	243	207	39.6
All (*n* = 100)	800	1381	1152	39.4

### Impact on Concerns

**FIGURE 2:**
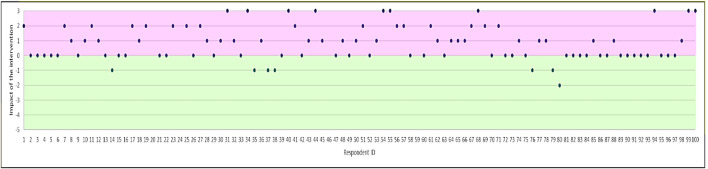
Magnitude of change in Concerns (difference in total score for the concerns subscale). Positive effect of the intervention is shown in purple (i.e. increase in concerns), and negative effect in green.

There was a significant increase in total Concerns scores after the intervention, with a mean difference in scores pre- and post-intervention of 0.930 (t = −7.214; *p* < 0.0001). Figure 2 shows the difference in total score for the Concerns subscale for each participant after the intervention. Concerns scores increased in over half (53%) of respondents, remained unchanged in 40%, and reduced in 7% of respondents.

Overall concerns shifted on average by 67%, with a similar effect size in both respondent groups. The percentage impact of the intervention on shifting concerns in each group is shown in Table 4. The statement “Taking a short course of antibiotics will not cause side effects” was associated with the largest shift in concerns (i.e. more participants agreed that even a short course of antibiotics can cause side effects, after exposure to the intervention).

**TABLE 4:** Percentage impact of the intervention on concerns.

Respondent group	Ideal score	Pre-intervention	Post-intervention	Impact
mTurk (*n* = 81)	729	607	688	66.4
Non-mTurk (*n* = 19)	171	154	166	70.6
All (*n* = 100)	900	761	854	66.9

### Impact on General perceptions of Antibiotics and AMR

**FIGURE 3:**
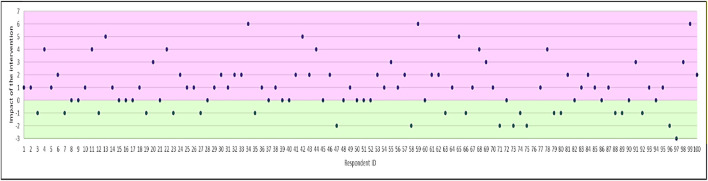
Magnitude of change in perceptions (difference in total score for the perceptions subscale). Positive effect of the intervention is shown in purple (i.e. increase in accuracy of perceptions of antibiotics and AMR).

There was a significant increase in total scores for general perceptions by 1.08 (*t* = −4.651; *p* < 0.0001), i.e., reduction in misconceptions post-intervention. The magnitude of change in perceptions scores is shown in Figure 3. Most respondents had positive shifts in their perceptions as illustrated by the large number of individuals with an increase in scores. Over half (58%) of participants demonstrated improved understanding of antibiotics and AMR, though beliefs did not change in 19% and shifted in the opposite direction in 23% of respondents.

Overall, general perceptions shifted by 30%, with a slightly greater impact seen on perceptions in the mTurk vs. non-mTurk group (Table 5). The statement “Using lower doses of antibiotics can help reduce risk of antibiotic resistance” had the largest increase, whilst the two reverse worded items had the smallest shifts: “Taking an antibiotic I don’t need will increase the risk of antibiotic resistance” and “Antibiotic resistance is when bacteria become resistant to the antibiotic”.

**TABLE 5:** Percentage impact of the intervention on general perceptions.

Respondent group	Ideal score	Pre-intervention	Post-intervention	Impact (%)
mTurk (*n* = 81)	1458	1160	1251	30.5
Non-mTurk (*n* = 19)	342	279	296	27.0
All (*n* = 100)	1800	1439	1547	29.9

### Aggregate Impact (Overall Effectiveness)

Table 6 shows the overall effect of the intervention, according to the direction of the changes seen post-intervention for each of the three subscales. Effects are split by group according to the type of recruitment method (mTurk vs. non-mTurk), and for both groups together.

**TABLE 6:** Overall effectiveness of the intervention.

mTurk	N	%	Non-mTurk	N	%	All	n	%
+++	25	30.9	+++	3	15.8	+++	28	28.0
++	20	24.7	++	5	26.3	++	25	25.0
+	17	21.0	+	4	21.1	+	21	21.0
0	11	13.6	0	4	21.1	0	15	15.0
-	3	3.7	-	3	15.8	-	6	6.0
--	3	3.7	--	0	0.0	--	3	3.0
---	2	2.5	---	0	0.0	---	2	2.0
	81	100.0		19	100.0		100	100.0

A total of 77% (+++ 31%, ++ 25%, + 21%) of all respondents (74.0% of mTurk respondents, 63.2% of non-mTurk respondents) had at least one positive shift (+++, ++ or +) in direction of either their necessity beliefs, concerns, or general perceptions of antibiotics and AMR after exposure to the intervention.

## Discussion

This is the first study to demonstrate that patient beliefs and general perceptions about antibiotics and AMR associated with inappropriate demand can be changed by a brief, tailored digital intervention. The intervention is the first to apply the NCF in a reverse way (NCF-R) with the aim of reducing unnecessary or undesired medication use. The intervention had a significant impact on beliefs, specifically reducing perceived necessity for antibiotics, increasing concerns about antibiotics and AMR, and improving the accuracy of general perceptions about antibiotics and AMR. The literature highlights several key factors which contribute to inappropriate use of antibiotics and demand for antibiotics by the public, even when not clinically needed. These factors include expectations and beliefs that antibiotics are an effective treatment for cold/flu symptoms, (Gaarslev et al., 2016) that antibiotics are needed when symptoms are prolonged or severe, (Cals et al., 2007; McNulty et al., 2013) and that use of antibiotics is associated with limited harm. (Bakhit et al., 2019) These beliefs can be considered as Necessity beliefs and Concerns, and general perceptions. This is in line with the NCF, which purports that medication use is influenced by an individual’s perceived personal need for a treatment, relative to concerns about potential negative effects of treatment. (Horne et al., 2013a) In the case of antibiotics, demand can be explained by a high perceived personal need for antibiotics, and a relative lack of concerns about potential harmful effects of antibiotics, including AMR.

The aim of the study was to shift beliefs about antibiotic necessity, and address the lack of concern about harmful effects of antibiotics, with the ultimate aim of reducing patient demand for unnecessary antibiotics. The study used a personalised logic algorithm to deliver brief behaviour change messages, tailored according to the specific beliefs of the individual. Our findings indicate that this novel application of the NCF-R was effective in shifting necessity beliefs, concerns and addressing perceptions in a positive direction to support more appropriate antibiotic use and awareness of AMR. There is potential for this “reverse NCF” approach to be applied to other health conditions which necessitate a reduction in demand for medication use (e.g. in addictions, or to support medication switches). The largest impact of the intervention appeared to be on antibiotic necessity and concerns, which is in-line with the original NCF, which identifies Necessity and Concerns to be predictors of medication use, but does not include general perceptions/ knowledge. (Horne et al., 2013b) The limited effect on the Perceptions domain may also be due to the high baseline scores. The changes in scores for Perceptions was also the least consistent, where 19% of participants had no change in perceptions and surprisingly 23% moved in the opposite direction. This could possibly be due to the reversed items “Taking an antibiotic I don’t need will increase the risk of antibiotic resistance,” and “Antibiotic resistance is when bacteria become resistant to the antibiotic” being confusing for the respondent; for both items, the items were worded to represent accurate statements, whilst the other statements were worded to reflect inaccurate beliefs. Whilst the initial rationale for including both positively and negatively worded items was to reduce response bias, (Sonderen et al., 2013) recent data suggests that including reversed items can confuse participants and influence questionnaire validity. (Sonderen et al., 2013; Chyung et al., 2018) Further validation and testing of this profiling questionnaire, with and without the reversed items, are needed.

Whilst the findings from this study are positive, there are several limitations. First, the intervention was only tested in using an online study in healthy participants using hypothetical scenarios to contextualise the intervention prior to intervention exposure. Whilst hypothetical scenarios are frequently used in the study of perceptions, beliefs, and attitudes, Timmermans et al. have expressed that the use of hypothetical scenarios may mean that decisions made by participants are not real, and so do not necessarily represent real-world phenomena in an accurate way. (Timmermans et al., 2008) Providing a more concrete hypothetical scenario, with lots of detail is preferable to the use of abstract questions about attitudes and perceptions. (Alexander and Becker, 1978) Future studies could test the effect of the intervention in patients in primary care at the time of presentation with cold/flu symptoms. The study was also conducted over a short timeframe, where the participants were followed-up immediately after intervention exposure . As such, it is unknown whether the effects seen in this intervention are sustainable, and if so, for how long.

Secondly, we did not assess the impact of the intervention on antibiotic consumption. Whilst we found statistically significant changes in belief scores, the changes are numerically modest. Behavioural science research has demonstrated many examples where small changes can have sizeable effects on behaviour, (Hauser et al., 2018) yet for antibiotic-seeking behaviour, it remains unknown whether these changes are clinically important and how this translates into behaviour. Further, the shifts in beliefs and perceptions are only proxy markers of effects, and it is not known whether these changes will translate into differences in future action (e.g. changes in antibiotic seeking and demand). Roope, et al. investigated factors which drive patient expectations of antibiotics and noted that 39% of people with low AMR awareness stated that the AMR information provided in the study would lead them to ask for antibiotics more often, a paradoxical consequence of AMR information campaigns (Roope et al., 2018) The study authors advocated for small scale testing for AMR education campaigns before wider adoption. Whilst our study has shown effectiveness in a small scale, future studies conducted in patient samples with longer-term follow-up are needed to see whether the intervention impacts on actual antibiotic demand and use in the future. Additionally, part of the process of interaction with patients also involves public health campaigns and education (Walker, 2019) How this individualised intervention interacts with this process and the rest of the health system, and whether the behaviour messages can be effective alone without the tailoring algorithm, needs further evaluation. Questions relating to implementation, scalability, cost-effectiveness, acceptability, and knowledge translation across different healthcare settings would need to be answered before the intervention could become part of routine practice.

The sample size of included participants was also small. Despite this, significant shifts in beliefs were observed showing that the intervention is efficacious in our sample. However, there may be sampling biases as the mTurk study sample may differ from the general population. To mitigate this, a sample of participants were recruited via ‘traditional’ recruitment methods, to act as a reference check for quality of respondent answers, though there are likely biases even with this non-mTurk group, as this was recruited via personal and research networks, and comprised health professionals, academic researchers, and behaviour change consultants which thus represents a selected group. Subgroup analysis for mTurk vs. non-mTurk respondents showed no significant differences, which provides some reassurance that mTurk responses were as accurate as responses from participants recruited via traditional means. This is in line with literature which demonstrates that mTurk sample responses are largely comparable to those collected via conventional methods (Huttner et al., 2014). However, as we could not collect any demographic data on the study sample, such as age, sex and ethnicity, and our non-mTurk sample is a selected group, further work is needed to determine the generalisability of our findings to other populations within and outside of the United Kingdom.

Importantly, this study is novel and differs from other AMS initiatives as the intervention was developed using behaviour change principles, an approach supported by evidence which shows that interventions grounded in behaviour change principles and health psychology are more effective (Norris et al., 2013; Gaarslev et al., 2016; Sanchez et al., 2016) This thus sets the foundation for future AMR and other health interventions where a reduction in medication use is desired, for example in medication switching or addictions.

## Conclusion

Patient beliefs, such as patient expectations and demand, are one of the key drivers of AMR. This is the first study to demonstrate that patient beliefs about antibiotics and AMR associated with inappropriate demand can be changed by a brief, tailored digital intervention that applies the NCF in novel way (i.e. in reverse–as the NCF-R). This intervention has potential to reduce inappropriate antibiotic use and AMR, and generates new knowledge on shifting beliefs about antibiotics and AMR. It sets the foundation for a pragmatic intervention that could potentially be integrated into practice to address the rising AMR threat globally, and applied to other situations where a reduction in medication use is desired.

## Data Availability

The raw data supporting the conclusions of this article will be made available by the authors upon reasonable request, without undue reservation.
