# Supplementary material for: Changing Patient and Public Beliefs About Antimicrobials and Antimicrobial Resistance (AMR) Using a Brief Digital Intervention
Source: Front Pharmacol. 2021 Mar 31;12:608971. doi: 10.3389/fphar.2021.608971 (PMC8045782; doi:10.3389/fphar.2021.608971)
Supplement: Supplementary file 1 [file DataSheet1.docx]

Appendices copyrighted and available on request to Professor Rob Horne

Appendix A - Profiling questionnaire © Professor Rob Horne

1. I need antibiotics because I feel really ill

2. I need antibiotics now because I am not getting any better

3. My body can tell me when I need antibiotics

4. I need antibiotics because I’ve been ill for more than 3 days

5. Getting an antibiotic is proof that I am ill

6. Only antibiotics can make me feel better

7. It is best to take an antibiotic to be on the safe side

8. I can stop the antibiotics once I feel better

9. I am concerned about antibiotic resistance

10. Antibiotics are harmless

11. Taking a short course of antibiotics will not cause side effects

12. There is not much I can do to help reduce antibiotic resistance

13. Using lower doses of antibiotics can help reduce risk of antibiotic resistance

14. Taking an antibiotic I don’t need will increase the risk of antibiotic resistance

15. Antibiotic resistance is when people become resistant to the bacteria

16. Antibiotic resistance is when bacteria become resistant to the antibiotic

17. New antibiotics will be developed in the future

Appendix B – Behaviour change messages © Professor Rob Horne

**Message 1**

It’s common sense that when you feel really ill you need an effective treatment – fast. Antibiotics are a powerful and effective treatment that can make people better quickly BUT there is a huge snag. They only work if the illness is CAUSED by bacteria. If you use antibiotics to treat something that isn’t caused by bacteria, they won’t work and can be harmful. So sometimes, it is best to hold off while your doctor tests if your illness is caused by bacteria or if you need an antibiotic at all. If it not caused by bacteria, other treatments will work better than antibiotics.

**Message 2**

It’s easy to feel you need antibiotics because you are feeling very unwell and not getting better. But antibiotics only work if your condition is caused by a bacterial infection. Most illnesses are NOT caused bacteria. Short-term illnesses such as colds and chills are usually caused by viruses not bacteria. In these cases, antibiotics won’t work and could make things worse. If your condition is not caused by bacteria, other treatments will be more effective.

**Message 3**

You may feel that you can tell when your body needs antibiotics – you may feel like you have had these symptoms before and antibiotics worked for you.  This is understandable.  However, many illnesses and conditions can give you the same symptoms, yet the cause is not always due to bacteria.  What can be confusing is your body reacts in the same way when it is sick even when the illness was not caused by bacteria. This can trick the body into thinking that it needs an antibiotic. This is especially true if you have had the symptoms before and an antibiotic seemed to work for you. But because bacterial illnesses can cause exactly the same symptoms as illnesses caused by other things (e.g., colds and viruses), it does not mean that antibiotics will work this time. Antibiotics only work if your illness is caused by bacteria, but your body can’t tell what is making it feel ill.  Your body can feel that it needs treatment but only special lab tests which your doctor can order can tell.

**Message 4**

It makes sense that if you’ve been ill for a while, you need treatment. But antibiotics may not be the best treatment for you. How long you have been ill is not related to whether or not you need antibiotics. Antibiotics only work if your illness is CAUSED by a bacterial infection. Most illnesses are not. For example, colds, coughs, chills and rashes are often caused by viruses. In these cases, antibiotics won’t work and may do more harm than good. It is better to match the treatment to the cause and avoid antibiotics when the illness is not caused by bacteria.

**Message 5**

Getting antibiotics may feel like a relief as it can make people feel like they are doing something to help the body get better when they’re ill.  But that may not be the case – having antibiotics when your illness isn’t caused by bacteria can do more harm than good.  The good news is there are other things you can do to help work out what’s wrong and how to get better.  For example, the doctor may order some lab tests to help find out what’s making you feel ill.

**Message 6**

Antibiotics can sometimes seem like magic – they can work to ‘fix’ the problem and make you better but only if they are taken for the right thing. But antibiotics can only work if your illness is caused by a bacterial infection, and most illnesses are not. Using antibiotics when you don’t have an illness caused by bacteria can be harmful. It can kill off the ‘good’ bacteria in your body, leaving you more vulnerable to other illnesses.

**Message 7**

When you are feeling ill and unwell, it is understandable to think that you need to take antibiotics to be on the safe side – similar to buying insurance. Antibiotics do not prevent you from getting sick; they only work if your illness is due to a bacterial infection. Most illnesses are NOT due to bacteria ad so antibiotics only work for a relatively small number of conditions, so taking them ‘just in case’ will not benefit you. In fact, it could be harmful to take antibiotics if you don’t need them.

**Message 8**

Antibiotic resistance is a big problem – if we don’t do something about this now, antibiotics will not in the future. This means people may get very ill or die from simple bacterial infections such as from skin scratches or wounds or routine surgery. Everyone can help to reduce this risk of antibiotic resistance. Antibiotics stop working when they are used in appropriately, for example, for illnesses not caused by bacteria. When this happens, bacteria are exposed to antibiotics which aren’t needed, making them stronger and ‘resistant’ (meaning the antibiotic can no longer kill them off – so called ‘superbugs’).

We can help prevent this by making sure antibiotics are only used when you need them, and taking antibiotics as prescribed by your doctor.

**Message 9**

Antibiotics work very well if they are given for the right condition. However, all medicines including antibiotics can have side effects, especially if taken when you don’t need them. Antibiotics can be harmful if taken when your body doesn’t have a bacterial infection, as they can kill off the ‘helpful’ bacteria in your body. This can cause problems like upset stomach and diarrhoea. Antibiotics can also have their own side effects such as causing rashes, or an upset stomach. It is important to only take antibiotics when you have an infection that’s caused by bacteria – check with your doctor first.

**Message 10**

All medicines, including antibiotics, can cause side effects. These side effects are unique for each individual and how long you take the antibiotic has very little to do with side effects. But there is a big variation between people. Some people find that they can take an antibiotic without getting a side effect, whilst others find that a particular antibiotic doesn’t ‘agree with them’ and they experience a side effect such as having loose bowels or stomach upset. It is difficult to predict this as some antibiotics are better suited to some people than others. If a particular antibiotic does not agree with you, then there is a risk of getting side effects even if you only have a short course. This is because getting a side effect depends more on the antibiotic, and how you react to it, rather than simply being about the ‘duration’ of the course.

**Message 11**

Antibiotics keep working in the background even when you feel well.  Even though you may feel better, the bacteria that made you unwell in the first place can still be in your body.  Making sure you finish your antibiotic course helps to make sure the bacteria causing your illness are completely removed from your body. This can also help to prevent antibiotic resistance.

**Message 12**

Antibiotic resistance is a real danger to us all. It occurs when bacteria become immune to the effects of antibiotics. If bacteria anywhere in the world are exposed to an antibiotic and survive, they can become resistant to that antibiotic. That means that that particular antibiotic will no longer work against them. Because they multiple very quickly, bacteria can pass on the resistance to other bacteria across the world in a very short period of time. The end result is that antibiotics which were once effective, no longer work. This means that people can become very ill or even die from simple infections because we no longer have effective antibiotics to treat the infection. The good news is everyone can help reduce the risk of resistance by making sure antibiotics are only used when you need them and taking them as prescribed by your doctor.

**Message 13**

Antibiotic resistance is more likely to happen if the dose of antibiotic is lower than it should be. That is because the bacteria are exposed to the antibiotic but survives. When this happens, it means the bacteria is resistant and that particular antibiotic will no longer work against them. Because they multiple very quickly, bacteria can pass on the resistance to other bacteria across the world in a very short period of time. The end result is that antibiotics which were once effective, no longer work. So, bacteria become resistant when the amount of antibiotic in the body isn’t enough. Taking the full dose that is prescribed is important to stop this resistance.

**Message 14**

Taking antibiotics when your body doesn’t need them can mean that the antibiotic may not work when you or your family really needs them.  This can happen because unnecessary use of antibiotics can kill off good bacteria or change bacteria into ‘resistant’ or super strong ones (called superbugs).  This can lead to infections that can’t be treated.  When this happens, routine infection like an infected cut or a bacterial sore throat, can become very dangerous if there are no antibiotics available to kill off bad bacteria.  The more antibiotics are used, the less effective they will be.  Therefore, it is important to always check with your doctor whether antibiotics are needed for your illness.

**Message 15**

Antibiotic resistance is something that happens to the bacteria NOT the person. If antibiotics are used when they are not needed, then they can become less effective against bacteria in the future. The bacteria become resistant to the antibiotic. This means that if the antibiotic is used in the future (for you or other people) it might not work at all. This is already happening for some antibiotics. If we do not stop it, by using antibiotics only when needed, we will end up with infections that we cannot treat.

**Message 16**

Antibiotic resistance can happen when bacteria in your body has been exposed to the wrong antibiotics. This can happen when you take antibiotics that are not needed, if the amount of antibiotic is not enough to kill off the bacteria such as when doses are missed, or antibiotics are stopped early.  You can prevent this from happening by checking with your doctor whether you need antibiotics or not for when you feel sick, and only taking the antibiotic as prescribed.

**Message 17**

It is hard for new antibiotics to be developed.  It can take 20-30 years for a new antibiotic to be made.  This means that when bacteria become resistant to antibiotics, there may not be antibiotics that work to kill them off.  If this happens common infections, such as strep throat, or routine surgery, such as hip replacements, may become life-threatening.  The good news is you can help prevent resistance from happening by using antibiotics appropriately – so only using antibiotics when prescribed by your doctor, finishing the course, and taking the dose that is advised by your health provider.
